# Application of Penalized Regression Techniques in Modelling Insulin Sensitivity by Correlated Metabolic Parameters

**DOI:** 10.1371/journal.pone.0141524

**Published:** 2015-11-06

**Authors:** Christian S. Göbl, Latife Bozkurt, Andrea Tura, Giovanni Pacini, Alexandra Kautzky-Willer, Martina Mittlböck

**Affiliations:** 1 Department of Gynecology and Obstetrics, Division of Feto-Maternal Medicine, Medical University of Vienna, Vienna, Austria; 2 Department of Internal Medicine III, Division of Endocrinology and Metabolism, Unit of Gender Medicine, Medical University of Vienna, Vienna, Austria; 3 Metabolic Unit, Institute of Neuroscience, National Research Council, Padova, Italy; 4 Center of Medical Statistics, Informatics and Intelligent Systems, Section for Clinical Biometrics, Medical University of Vienna, Vienna, Austria; University Medical Center Göttingen, GERMANY

## Abstract

This paper aims to introduce penalized estimation techniques in clinical investigations of diabetes, as well as to assess their possible advantages and limitations. Data from a previous study was used to carry out the simulations to assess: a) which procedure results in the lowest prediction error of the final model in the setting of a large number of predictor variables with high multicollinearity (of importance if insulin sensitivity should be predicted) and b) which procedure achieves the most accurate estimate of regression coefficients in the setting of fewer predictors with small unidirectional effects and moderate correlation between explanatory variables (of importance if the specific relation between an independent variable and insulin sensitivity should be examined). Moreover a special focus is on the correct direction of estimated parameter effects, a non-negligible source of error and misinterpretation of study results. The simulations were performed for varying sample size to evaluate the performance of LASSO, Ridge as well as different algorithms for Elastic Net. These methods were also compared with automatic variable selection procedures (i.e. optimizing AIC or BIC).We were not able to identify one method achieving superior performance in all situations. However, the improved accuracy of estimated effects underlines the importance of using penalized regression techniques in our example (e.g. if a researcher aims to compare relations of several correlated parameters with insulin sensitivity). However, the decision which procedure should be used depends on the specific context of a study (accuracy versus complexity) and moreover should involve clinical prior knowledge.

## Introduction

Impaired insulin sensitivity is considered as an important risk factor for metabolic disorders and of particular importance in the pathogenesis of type 2 diabetes [1]. Several indices containing measurements derived from the oral glucose tolerance test (OGTT), biomarkers and parameters of body composition have been proposed to evaluate insulin sensitivity in humans. Surrogate indices of insulin sensitivity are of clinical importance, as its direct evaluation by the hyperinsulinemic euglycemic clamp (“gold standard”) or alternatively the frequently sampled intravenous glucose tolerance test (FSIGT, sometimes referred as the “silver standard”) are rather time and cost intensive examinations [2].

In clinical research settings of diabetes, multiple linear regression is often used to predict insulin sensitivity (derived from clamp or FSIGT data as response variable *Y*) by several independent variables (*X* ^*T*^ = (*x*_1_,…*x*_*k*_)): e.g. OGTT measurements (including repeated measurements of glucose, insulin and C-peptide for example), biochemical markers derived from fasting and postprandial state or parameters of body composition. Thereby, linear regression might be used to conduct a prediction model for insulin sensitivity or to select predictors out of a set of variables with accurate predictor estimates (e.g. to select the most relevant OGTT measurements and their best measurement time). However, as the sample size of the evaluation cohorts might be sparse in several cases (due to the limited availability of clamp or FSIGT data) and explanatory variables are supposed to be highly correlated in clinical scenarios of metabolic disorders, statistical limitations of the linear regression approach, such as collinearity and overfitting have to be considered. These limitations might have large influence on parameter estimates derived from “traditionally used” automatic variable selection procedures (such as forward, backward, or stepwise-backward (a combination of forward and backward selection)) and therefore on the reproducibility of results, particularly if a validation cohort with adequate sample size is missing [3].

By introducing a slight bias into the model estimation, penalized estimation techniques were proposed to reduce the variance of estimates and hence to improve prediction [4]. Particularly three methods achieved high popularity: Ridge (shrinks the sum of squares of regression coefficients toward zero) [5, 6], LASSO (Least Absolute Shrinkage and Selection Operator, shrinks the sum of absolute values of regression coefficients toward zero) [7, 8] as well as the elastic net (a hybrid of Ridge and LASSO) [9]. These strategies were primarily developed to deal with high dimensional correlated data sets (i.e. DNA-microarray/genomic studies) where they showed a good performance [4, 10]. More recently, penalized estimation techniques were also discussed to provide reasonable results in low dimensional data scenarios [11, 12].

Therefore, this paper aims to introduce variable shrinkage strategies in clinical investigations of diabetes, as well as to assess their possible advantages and limitations. Moreover, comparisons with traditionally used sequential selection procedures should be assessed in different simulation scenarios. Particular focus should be placed on the prediction of insulin sensitivity with correlated covariates to investigate: a) which strategy shows the lowest prediction error (of importance when the research question is to create a new surrogate index of insulin sensitivity) and b) which procedure gives the most accurate estimation of regression coefficients (of importance when the specific relation between an independent variable and insulin sensitivity should be evaluated) including the correct estimation direction and variable selection probabilities (power and type 1 error). Data from a real clinical investigation was used for illustration purposes.

## Clinical Data Example: Vienna Post Gestational Diabetes Data

A description of the Vienna Post-Gestational Diabetes Project was reported elsewhere (e.g. [13, 14]). The diabetes data contains parameters of body composition (BMI, waist and hip circumference) as well as OGTT measurements (blood samples of glucose, insulin, C-peptide, proinsulin and amylin were taken at fasting as well as frequently at 10, 20, 30, 60, 90, 120, 150 and 180 minutes after ingestion of 75 g glucose) of 110 females after pregnancy with gestational diabetes. FSIGT derived insulin sensitivity index (SI) by minimal model analysis according to Pacini et al. [15] was available in 102 subjects. All subjects were recruited 3 to 6 month after index pregnancy between 1999 and 2002. The study was approved by the Ethics Committee of the Medical University of Vienna and performed in accordance with the Declaration of Helsinki. All participants gave written informed consent. Patient related information was anonymized and de-identified prior to analysis.

## Methods of Estimation

### Linear regression

The ordinary least squares regression (OLS), aims to predict future cases of *y* by a list of known explanatory variables (regressors). The linear model is defined by

$$y_{i}=\beta_{0}+\beta_{1}\cdot x_{i1}+\ldots +\beta_{k}\cdot x_{ik}+\varepsilon_{i}$$

where *y*_*i*_ denotes the dependent variable of the *i* ^*th*^ patient (*i* = 1,…,*n*) and *x*_*i*1_,…,*x*_*ik*_ denoting the corresponding *k* explanatory variables; *β*_0,_ *β*_1_,…,*β*_*k*_ are the regression coefficients; *β*_0_ denotes the intercept and *ε*_*i*_ denotes the normally distributed model error with an expected value of zero and a residual variance *σ* ^2^. Interaction terms were not considered in this study. In order to minimize the residual sum of squares the minimization problem is described as:

$$\sum_{i=1}^{n}\varepsilon_{i}^{2}=\left\{\sum_{i=1}^{n}{\left(y_{i}-\beta_{0}-\sum_{j=1}^{k}x_{ij}\beta_{j}\right)}^{2}\right\}\to \min.$$

The different methods, which are suggested to improve the naïve OLS estimation ${\hat{\beta }}^{OLS}$ (i.e. sequential variable selection and penalized estimation strategies), are outlined in the following.

### Sequential variable selection strategies

For this report, we used a stepwise-backward variable selection procedure, optimizing two different parameters of entropy:

1. Akaike’s Information Criterion (AIC) [16]:
  $$AIC=-2\cdot \ln\left(\frac{RSS}{n}\right)\cdot \left(-\frac{n}{2}\right)+2k$$
  where *RSS* denotes the residual sum of squares and *n* denotes the total number of observations.
2. Schwarz’s Bayesian Information Criterion (BIC) [17]:
  $$BIC=-2\cdot \ln\left(\frac{RSS}{n}\right)\cdot \left(-\frac{n}{2}\right)+k\cdot \ln(n).$$

The first part of AIC and BIC is also called deviance and gives information on the model fit. The second term is a penalty for model complexity, depending on the number of parameters fitted.

When sequential selection strategies with AIC or BIC (denoted sAIC and sBIC) are used then selection criterion values with and without a candidate variable are calculated. The model with the lower criterion value is preferable and correspondingly the candidate variable is included or excluded. For AIC, the candidate variable is included if the difference in deviances with and without the respective predictor (i.e. model Χ^2^ value) exceeds two times the difference of parameters fitted (difference in degrees of freedom) of the two models. Hence, for a linear (or binary) predictor the Χ^2^ value has to exceed 2, comparable to a p-value of 0.157 (if the F-statistic is used for selection) [18, 19]. In contrast, BIC penalizes the model deviance by the product of the number of covariates and the natural logarithm of the number of observations (n). It can be shown, that in case of n>e^2^ (≈7) the penalty by BIC is larger as compared to AIC (if the number of covariates are comparable) [18]. BIC tends to select the correct model with infinite sample size (n → ∞), whereas AIC tends to select too complex models with n≥8. Thus, the optimal choice between AIC and BIC is not generally clear [4].

### Variable shrinkage by Ridge

If prediction vectors are not orthogonal it was proposed that introducing a slight bias would substantially decrease the variance and thus improve predictions [5]. In case of a linear regression scenario, Ridge estimation can be considered by the following minimization problem [4]:

$${\hat{\beta }}^{ridge}=\underset{\beta }{\arg\;\min}\left\{\sum_{i=1}^{n}{\left(y_{i}-\beta_{0}-\sum_{j=1}^{k}x_{ij}\beta_{j}\right)}^{2}+\lambda_{2}\sum_{j=1}^{k}\beta_{j}^{2}\right\}.$$

Or with another notation:

$${\hat{\beta }}^{ridge}=\underset{\beta }{\arg\;\min}\sum_{i=1}^{n}{\left(y_{i}-\beta_{0}-\sum_{j=1}^{k}x_{ij}\beta_{j}\right)}^{2},\;\text{under the constraint}:\;\sum_{j=1}^{k}\beta_{j}^{2}\leq s.$$

There is a 1:1 correspondence between the complexity parameters *λ*_2_ and *s*, controlling the amount of shrinkage of the regression coefficients toward zero (whereby *β*_0_ is not penalized). Ridge is based on the sum of squares of the regression coefficients and results in a proportional shrinkage of parameter estimates (but not in exclusion of variables) [4].

### Variable shrinkage and selection by LASSO

Tibshirani introduced the LASSO method with the advantage that it also performs variable selection in addition to shrinkage [7]. In contrast to Ridge, LASSO uses the sum of absolute values of regression coefficients for penalization of model complexity. It shrinks each coefficient toward zero by a constant factor, truncating at zero. Consequently variables with zero-truncated coefficients are excluded from the model [4]:

$${\hat{\beta }}^{LASSO}=\underset{\beta }{\arg\;\min}\left\{\sum_{i=1}^{n}{\left(y_{i}-\beta_{0}-\sum_{j=1}^{k}x_{ij}\beta_{j}\right)}^{2}+\lambda_{1}\sum_{j=1}^{k}\left|\beta_{j}\right|\right\}.$$

Or with another notation:

$${\hat{\beta }}^{LASSO}=\underset{\beta }{\arg\;\min}\sum_{i=1}^{n}{\left(y_{i}-\beta_{0}-\sum_{j=1}^{k}x_{ij}\beta_{j}\right)}^{2},\;\text{under the constraint}:\;\sum_{j=1}^{k}{\left|\beta \right|}_{j}\leq t.$$

Again, the amount of shrinkage of |*β*_*j*_| is controlled by a tuning parameter *λ*_1_ or *t*: If *t* is chosen to be larger than the sum of the absolute values of the OLS estimates then the estimates proposed by LASSO are comparable to those provided by OLS. For sufficiently small *t* (large *λ*_1_) parameter estimates for explanatory variables might be shrunken to zero [4] and the variables are thus excluded from the model.

## Variable shrinkage and selection by Elastic Net

The Elastic Net (Enet) was more recently developed by Zou and Hastie [9] as a combined variable shrinkage and variable selection procedure for scenarios with highly correlated predictors, as LASSO was suggested to be inferior to Ridge in nonorthogonal scenarios. The minimization problem of Enet is defined as [4, 9]:

$${\hat{\beta }}^{Enet}=\underset{\beta }{\arg\;\min}\left\{\sum_{i=1}^{n}{\left(y_{i}-\beta_{0}-\sum_{j=1}^{k}x_{ij}\beta_{j}\right)}^{2}+\lambda \sum_{j=1}^{k}\left(\alpha \beta_{j}^{2}+\left(1-\alpha \right)\left|\beta_{j}\right|\right)\right\}.$$

Where *λ* = *λ*_1_ + *λ*_2_ and *α* determines a “mix of penalties” and is calculated as [4, 9]:

$$\alpha =\frac{\lambda_{2}}{\lambda_{1}+\lambda_{2}}.$$

According to Waldron et al., three different methods of choosing the tuning parameters were assessed [10]: a) first optimizing *λ*_1_ (while keeping *λ*_2_ at zero) followed by optimizing *λ*_2_ (Enet 1); b) first optimizing *λ*_2_ followed by optimizing *λ*_1_ (Enet 2); c) optimizing *λ*_1_ and *λ*_2_ simultaneously (Enet 3).

### Cross-validation for optimizing the tuning parameter

Cross-validation is a widely used technique to assess the expected generalization error and is particularly established in estimating the shrinkage parameter. Particularly K-fold cross-validation (with K = 5 or K = 10) has been proposed to give appropriate results [4]: The data is split into K parts of equal sample size and the respective model is fitted to K-1 parts of the data. The prediction error is estimated by prediction of the remaining part. This procedure is repeated K times. The value for the tuning parameter with the smallest prediction error is preferred.

### Software

Calculations were performed by using R (V3.0.1). Stepwise-backward variable selection was performed by the “MASS” package by using the stepAIC function where the option k = 2 or k = log(n) gives the multiple of the number of degrees of freedom used for the penalty and result thus in AIC or BIC, respectively [20]. Variable shrinkage (Ridge, LASSO, Enet 1 and Enet 2) was performed using the “penalized” package [21, 22]. The “pensim” package was used for simultaneous optimization of *λ*_1_ and *λ*_2_ (Enet 3) [10]. The shrinkage parameters were assessed by 10-fold cross validation.

## Design of Data Simulations

### Overview

All simulations were performed according to the data of the Vienna Post-Gestational Diabetes Project (particularly parameter estimates and covariance structure were obtained from this study). Thereby three scenarios (detailed description is provided below) were investigated to assess which method of estimation shows the lowest prediction error of the final model in a set of a large number of variables (k = 20) with high collinearity (scenario A) as well as to assess which procedure gives the most accurate estimate of regression coefficients in a scenario with k = 9 variables with small unidirectional coefficient estimates and moderate collinearity (scenario B). Scenario C was comparable to scenario B, however, four additional variables with zero effect were included. A total of m = 5000 simulations were performed for varying sample sizes (n = 50, n = 110 (comparable to the sample size of the original data), and n = 500). The accuracy of different model building strategies was described as bias (estimated by mean $\hat{\beta }-\beta$, where $\hat{\beta }$ denotes the estimated effects and *β* the "true" effect i.e. the specific effect used in simulation study) and root mean square error (RMSE) of $\hat{y}-y$, where $\hat{y}$ is the estimated value in m = 5000 simulations.

The amount of collinearity was expressed by calculating the Variance Inflation Factor (VIF) for an explanatory variable *X*_*j*_, which is expressed as [23]:

$$VIF_{j}=\frac{1}{\left(1-R_{j}^{2}\right)}.$$

$R_{j}^{2}$ is the multiple *R* ^2^ of a linear model, with *X*_*j*_ as dependent variable regressed by the remaining variables in the original data set. VIF = 1 corresponds to an orthogonal system. The occurrence of collinearity is indicated if VIF exceeds 5 or 10, respectively [24].

### Design of simulation scenario A

The primary focus of this scenario was to evaluate the prediction error of the final model (i.e. prediction error of *Y*). This might be of interest if a researcher is focused on the prediction of insulin sensitivity.

**Fig 1:**
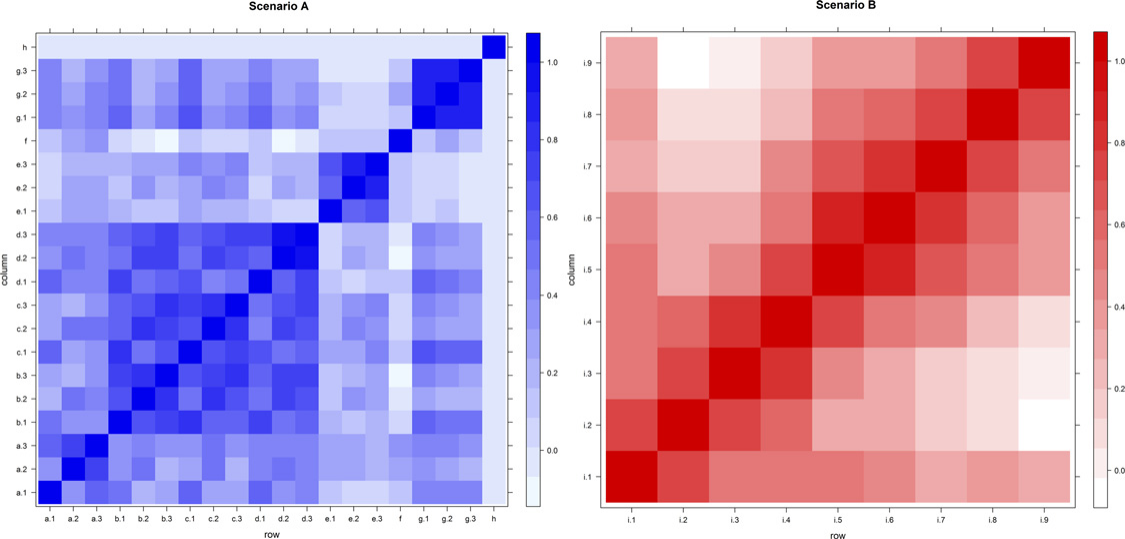
Visualisation of correlation structure of k = 20 explanatory variables (Scenario A) and k = 9 variables (Scenario B). Darker plots indicate higher (positive) correlations between variables.

Therefore, multiple linear regression simulations were conducted, including a number of k = 20 variables. The model coefficients (which were used as the “true effects” for this scenario) are provided in Table 1 together with corresponding VIF. The correlation structure of predictor variables, which was used for the simulations was based on the clinical data example and is visualized in Fig 1A. An uncorrelated noise variable was included to assess the amount of type 1 error (i.e. selection probability of a variable with zero effect). Moreover, a Gauss distributed random error variable with *σ* = 0.4 as well as an intercept of 1.45 were included into the formula, in accordance with the original data.

**Table 1:** Design of simulation scenario A.

Variable (scenario A)	a.1	a.2	a.3	b.1	b.2	b.3	c.1	c.2	c.3	d.1
β	0.03	-0.11	-0.09	0.15	-0.12	-0.20	-0.27	0.30	-0.12	0.01
VIF	2.57	4.69	4.87	7.27	18.9	14.2	10.9	33.3	27.0	4.99
Variable (scenario A)	d.2	d.3	e.1	e.2	e.3	f.1	g.1	g.2	g.3	h
β	-0.02	0.04	0.04	-0.18	0.15	0.08	-0.03	-0.09	-0.06	0.00
VIF	25.3	32.1	2.79	8.70	12.0	1.40	11.0	6.24	6.84	1.00

True regression coefficients (*β*) and Variance Inflation Factor (VIF) for k = 20 variables explaining insulin sensitivity. They were calculated to estimate insulin sensitivity (i.e. ln[SI+1]) by OLS: a.1(fasting glucose), a.2 (120’ post load glucose), a.3 (AUC glucose*), b.1(fasting insulin*), b.2 (120’ post load insulin*), b.3 (AUC insulin*), c.1(fasting C-peptide**), c.2 (120’ post load C-peptide**), c.3 (AUC C-peptide*), d.1(fasting proinsulin*), d.2 (120’ post load proinsulin*), d.3 (AUC proinsulin*), e.1(fasting amylin***), e.2 (120’ post load amylin***), e.3 (AUC amylin*), f (age), g.1 (BMI*), g.2 (waist circumference*), g.3 (hip circumference*), h (uncorrelated noise variable)

* ln[x]

** ln[x+1]

*** sqrt[x]

In accordance with the real data example, the mean-adjusted *R* ^2^ was 0.52 (for n = 50) and 0.53 (for n = 110 and n = 500) for the OLS estimation of data simulations, respectively.

For additional insight to the dependence of explained variation and model selection behavior the simulations were repeated for n = 500 with varying *σ* of the error variable with 0.2, 0.6, and 0.8 corresponding to a mean-adjusted *R* ^2^ of 0.82, 0.34 and 0.22, respectively.

## Design of simulation scenario B and scenario C

These scenarios were proposed to assess the behaviour of shrinkage and selection strategies if the research question is rather focused on an accurate estimation of regression coefficients (scenario B) or to pic-up important variables out of a set of correlated measurements (scenario C). This might be of relevance if a researcher is aiming to study the association of measurements of a parameter (e.g. glucose) at multiple time points during the OGTT with the degree of insulin sensitivity or to pic-up the most relevant time-point(s) for clinical purposes.

Therefore, a number of k = 9 variables were generated for linear regression simulations to predict *Y*. The correlation structure used for the simulations was based on plasma glucose measurements of the diabetes data example (fasting, 10’, 20’, 30’, 60’, 90’, 120’, 150’, 180’ after oral glucose load: i.1-i.9) as provided in Fig 1B. The VIF for each explanatory variable in the original data were: 3.06 (i.1), 3.94 (i.2), 4.99 (i.3), 6.57 (i.4), 7.61 (i.5), 7.06 (i.6), 4.36 (i.7), 4.51 (i.8), 2.76 (i.9). Moreover a Gaussian distributed random error variable with *σ* = 0.3 as well as an intercept of 1.45 was included. The true regression coefficients *β* (which should be predicted by various methods) were set to -0.025 for all variables to achieve small unidirectional effects as expected for metabolic studies (scenario B).

Scenario C was comparable to scenario B, however, four regression coefficients (i.2, i.3, i.8, i.9) were set to zero (noise variables) and *σ* was 0.15. For both scenarios, the correlation matrix was obtained from the original data set and is visualized in Fig 1.

The mean-adjusted *R* ^2^ were 21.3% (n = 50), 21.7% (n = 110) and 21.9% (n = 500) for the OLS estimation in scenario B as well as 30.7% (n = 50), 31.1% (n = 110) and 31.3% (n = 500) for the OLS estimation in scenario C, respectively.

## Results

### Descriptive analysis of tuning parameters

The distribution of cross-validated tuning parameters for various scenarios and sample size examples are given in Table 2: In all scenarios and sample sizes the *λ*_2_ penalty dominated over *λ*_1_ in Enet 2 and Enet 3 and hence was very close to Ridge. In contrast, *λ*_1_ penalty of Enet 1 was very close to LASSO, whereas *λ*_2_ was markedly different from zero, but still smaller as compared to *λ*_2_ penalties of other methods. Notably, the amount of shrinkage decreased with ascending sample size in scenario A, whereas the amount of penalty rather increased with sample size in scenarios B and C.

**Table 2:** Distribution of the shrinkage parameters in different scenarios and sample sizes.

	Ridge	LASSO	Enet 1		Enet 2		Enet 3	
	λ2	λ1	λ1	λ2	λ1	λ2	λ1	λ2
**Scenario A**								
**n = 50**	89.4 (64.2–117.9)	3.57 (2.66–4.63)	3.59 (2.68–4.61)	21.1 (4.91–43.9)	0.00 (0.00–0.57)	89.2 (64.0–118.2)	0.00 (0.00–0.83)	78.7 (41.0–112.0)
**n = 110**	69.2 (13.9–104.8)	3.70 (2.57–5.07)	3.73 (2.57–5.12)	19.9 (3.63–42.1)	0.37 (0.00–1.47)	69.6 (40.1–102.4)	0.04 (0.00–1.21)	55.6 (13.9–104.8)
**n = 500**	4.99 (3.20–8.09)	0.68 (0.41–1.48)	0.69 (0.42–1.47)	1.22 (0.00–4.35)	0.10 (0.00–0.60)	4.98 (3.13–8.06)	0.00 (0.00–0.42)	3.79 (1.07–7.88)
**Scenario B**								
**n = 50**	83.2 (54.5–128.2)	1.90 (1.28–2.91)	1.89 (1.26–2.88)	31.1 (6.45–69.0)	0.00 (0.00–0.22)	83.2 (54.7–129.4)	0.00 (0.00–0.80)	79.6 (41.1–132.4)
**n = 110**	99.3 (72.7–131.3)	1.75 (1.25–2.47)	1.77 (1.27–2.50)	48.4 (15.4–83.6)	0.00 (0.00–0.06)	100.6 (72.0–131.8)	0.00 (0.00–0.19)	94.6 (53.0–124.9)
**n = 500**	191.8 (149.0–227.9)	1.97 (1.38–2.63)	1.97 (1.38–2.63)	149.1 (88.8–193.3)	0.00 (0.00–0.00)	191.7 (150.5–228.1)	0.00 (0.00–0.01)	178.6 (112.1–223.0)
**Scenario C**								
**n = 50**	60.0 (39.9–85.8)	0.94 (0.66–1.32)	0.94 (0.67–1.32)	18.2 (3.41–38.7)	0.00 (0.00–0.19)	59.8 (40.3–85.4)	0.00 (0.00–0.42)	50.0 (18.7–80.2)
**n = 110**	68.6 (47.8–90.5)	1.03 (0.75–1.39)	1.03 (0.74–1.39)	27.7 (9.49–51.5)	0.00 (0.00–0.33)	68.8 (47.8–90.8)	0.00 (0.00–0.49)	57.5 (19.2–87.0)
**n = 500**	104.7 (74.2–135.1)	1.67 (1.20–2.12)	1.65 (1.18–2.12)	52.1 (25.4–84.1)	0.36 (0.00–0.97)	104.4 (74.3–135.6)	0.31 (0.00–0.96)	103.7 (38.7–126.1)

Data represent median values as well as 1^st^ and 3^rd^ quartiles of the cross-validated tuning parameters for n = 50, 110 and 500 and both simulation scenarios. Ridge method (Ridge); least absolute shrinkage and selection operator (LASSO); elastic net L1-L2 (Enet 1), elastic net L2-L1 (Enet 2), elastic net L1+L2 (Enet 3).

### Scenario A

**Fig 2:**
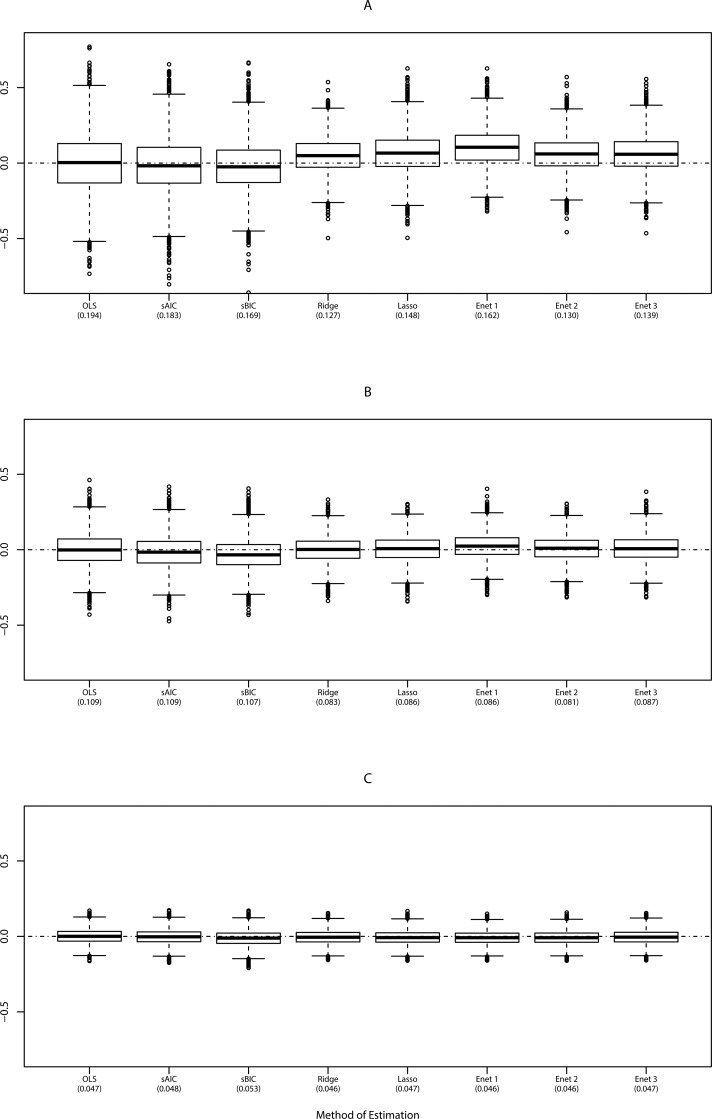
Scenario A: Box-plots show the distribution of the prediction errors (observed and estimated *y*), when using different methods of estimation in scenario A with n = 50 (A), n = 110 (B) and n = 500 (C). RMSE is given in parentheses. sAIC and sBIC refers to stepwise-backward variable selection with AIC and BIC.

As shown in Fig 2 only moderate differences were observed between the methods in terms of bias and prediction error. Compared to the OLS method sequential selection strategies (particularly optimizing BIC (sBIC)) as well as penalized regression methods showed slightly improved RMSE in case of low sample size (n = 50). Particularly, the most improved prediction error of *Y* was observed for Ridge (RMSE = 0.1270). Also in the scenario with n = 110 penalized estimation methods showed only a moderate benefit compared to OLS and sequential selection strategies. While the RMSE was considerably improved with increasing sample size, the effect of sequential selection as well as variable shrinkage strategies in relation to the naïve OLS estimation was attenuated.

**Fig 3:**
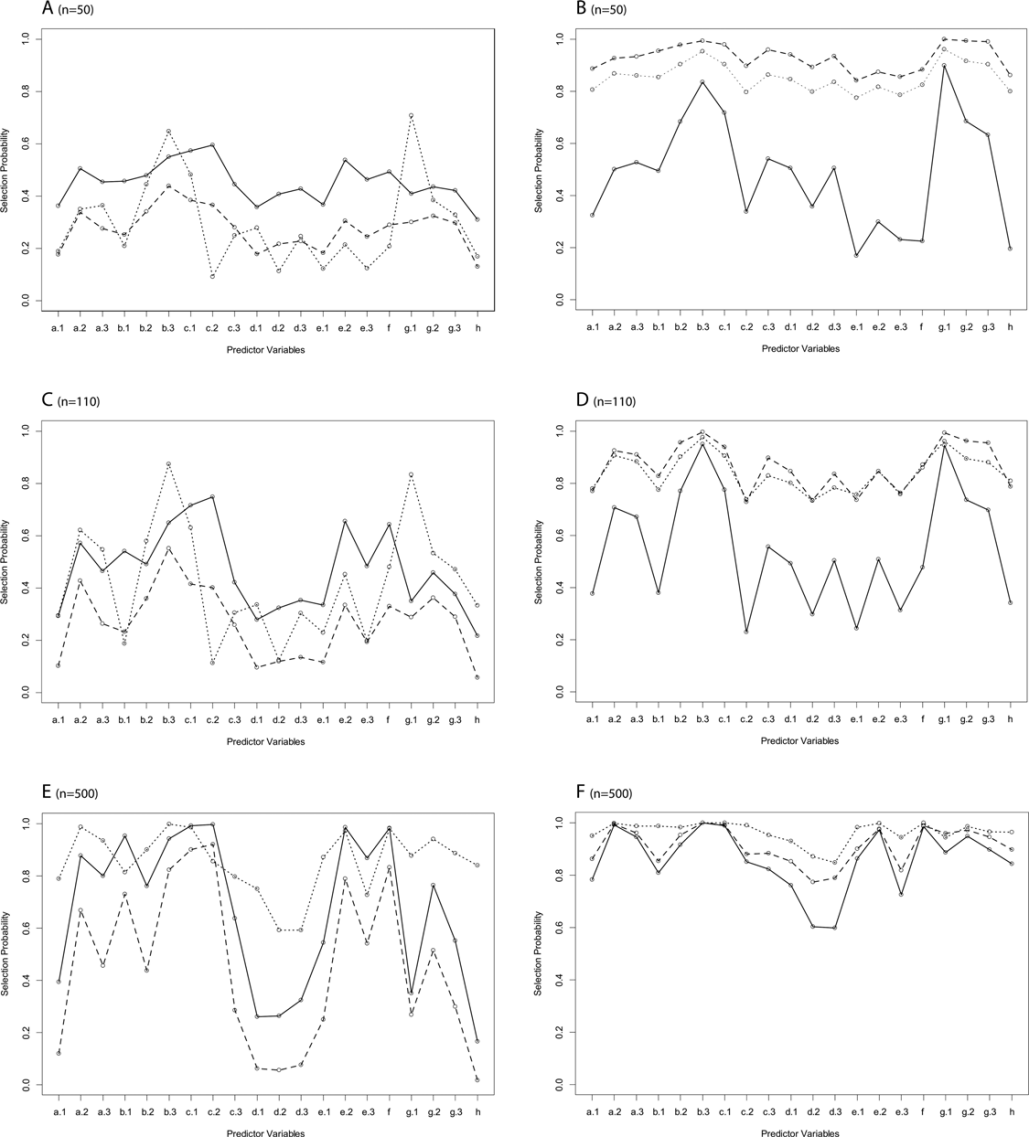
Scenario A: Selection probability for different variable selection methods in scenario A. n = 50 (A, B), n = 110 (C, D) and n = 500 (E, F). A, C, E: solid line for sAIC, dashed line for sBIC, dotted line for LASSO; B, D, F: solid line for Enet 1, dashed line for Enet 2, dotted line for Enet 3. Noise predictor variable is h.

However, we observed strong differences in the number of selected variables (Fig 3): LASSO performs sparser models as compared to other shrinkage strategies, but tended to include more variables into the model, if sample size was increased. In contrast to LASSO, sequential selection strategies tended to select fewer variables with increasing sample size. Notably, sBIC always selected sparser models than optimizing AIC (sAIC) and had also slightly smaller RMSE than sAIC for small to medium large sample size (n = 50, n = 110). In all situations Enet 2 and Enet 3 selected larger models than other strategies, whereas the selection profile of Enet 1 was almost comparable to LASSO. Accordingly, LASSO (16.9%), Enet 1 penalization (19.4%) and moreover sBIC (12.9%) showed a lower type 1 error (i.e. the probability of selecting the noise variable "h") in the low sample size scenario (n = 50) as compared to sAIC (30.9%), Enet 2 (86.2%) but also the Enet 3 (79.9%) algorithm. However, type 1 error increased strongly with growing sample size particularly for shrinkage techniques: LASSO (33.2% and 84.0%) and Enet 1: (34.0% and 84.3%) for n = 110 and n = 500, respectively. To further investigate this very high type 1 error, observed even in the large sample size scenario, we repeated simulations for n = 500 with varying *σ* of the error variable with 0.2, 0.6, and 0.8 corresponding to a mean-adjusted *R* ^2^ of 0.82, 0.34 and 0.22, respectively. The amount of shrinkage increased with increasing *σ* (median *λ*_1_ was 0.17, 6.3 and 11.5) and type 1 error, that is false positive selection of the nuisance variable h decreased from 96% to 60% and 50%, respectively.

### Scenario B

**Fig 4:**
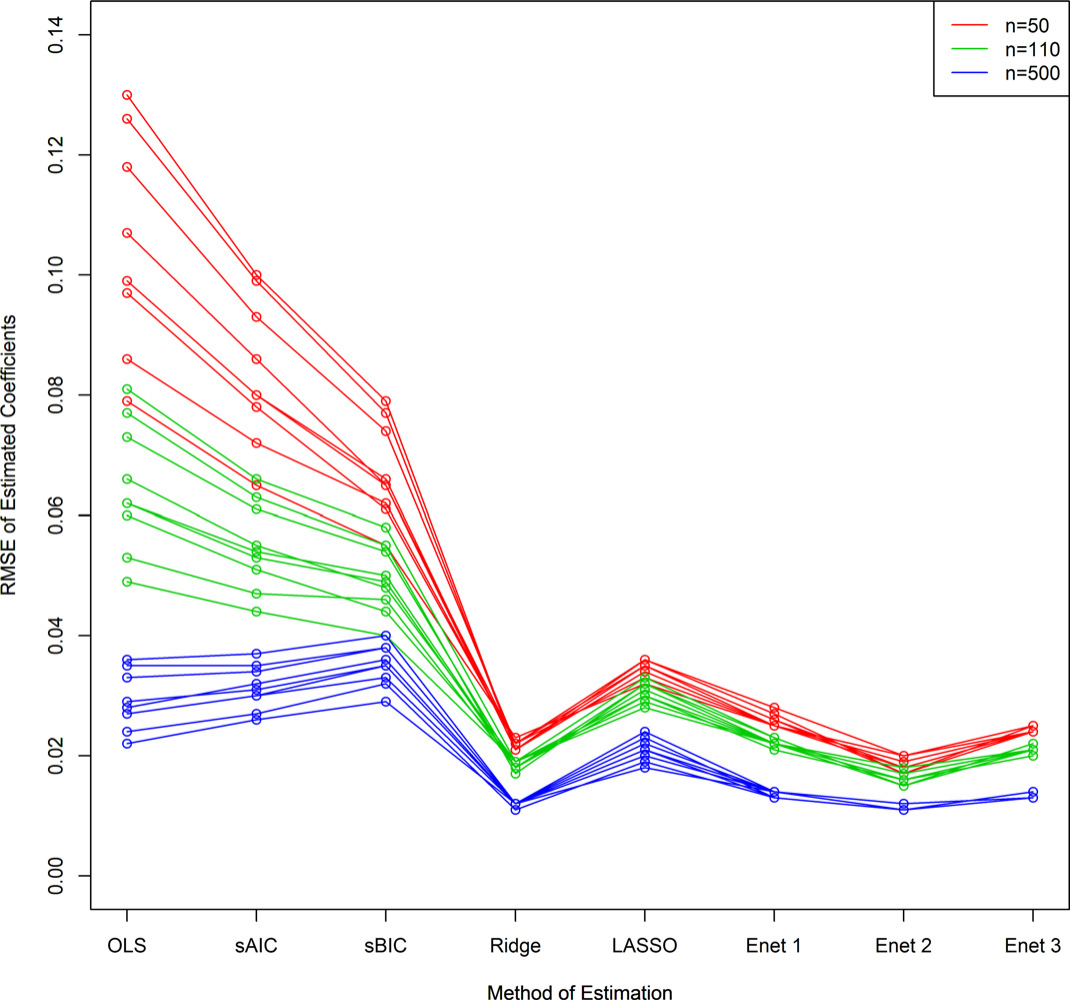
Scenario B: RMSE for the 9 regression coefficients using different model building strategies and varying sample sizes.

**Fig 5:**
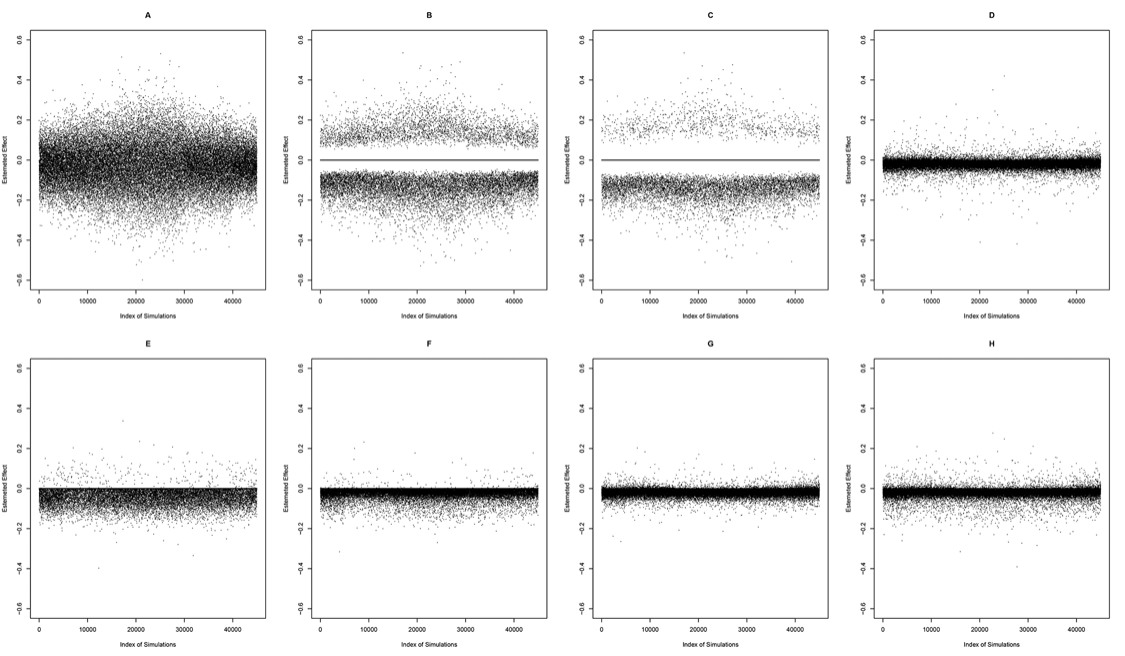
Scenario B: Estimated effect for the 9 regression coefficients (true effect = -0.025) using different model building strategies in 5000 simulated datasets (n = 50). OLS (A), sAIC (B), sBIC (C), Ridge (D), LASSO (E), Enet1 (F), Enet2 (G), Enet3 (H).

As illustrated in Fig 4 shrinkage strategies showed considerably improved RMSE as compared to sAIC or sBIC in all sample size scenarios and thus tended to give a more accurate estimate of regression coefficients. Of note, Ridge and Enet algorithms showed the most improved estimation (lowest RMSE for $\hat{\beta }$). The probability of each method to select the regression coefficient with the wrong sign (related interpretation to a type 3 error) is given in Table 3, demonstrating advantages of LASSO as well as Enet 1 in scenarios with low sample size, whereas the probability that a variable was not selected was even lower as compared to sAIC and sBIC. Variable selection by optimizing sBIC obtained the wrong sign less often as compared to sAIC but was still inferior to LASSO type penalization if the sample size was low. As assumed, the naïve OLS estimation showed clearly worse results as compared to all other methods. Estimated effects for all nine regression coefficients in m = 5000 simulations is visualized in Fig 5 (for the n = 50 case). sAIC and sBIC excluded particularly smaller effects, whereas large estimated coefficients (regardless of their sign) were not penalized in contrast to shrinkage procedures. This might cause a potential limitation for the use of these methods in the context of metabolic studies with primarily correlated predictors with small effects, in particular if a researcher is interested to compare different effect sizes.

**Table 3:** Percentage of wrong sign for the estimated regression coefficient (scenario B).

	OLS	Ridge	sAIC	sBIC	LASSO	Enet 1	Enet 2	Enet3
**i.1** (n = 50)	38.5 (0.0)	8.7 (0.0)	6.2 (67.3)	1.6 (80.5)	0.7 (56.5)	0.6 (41.3)	4.4 (8.5)	4.4 (21.3)
**i.2**	40.6 (0.0)	11.8 (0.0)	8.4 (66.5)	2.6 (80.8)	0.9 (70.3)	0.7 (54.0)	6.9 (10.2)	6.4 (23.3)
**i.3**	39.9 (0.0)	9.6 (0.0)	7.4 (66.3)	2.0 (80.1)	0.7 (67.2)	0.4 (49.7)	4.7 (9.5)	5.0 (22.5)
**i.4**	42.0 (0.0)	6.2 (0.0)	7.8 (66.9)	2.4 (78.7)	0.5 (61.9)	0.3 (40.5)	2.3 (7.9)	2.9 (21.8)
**i.5**	42.6 (0.0)	5.3 (0.0)	9.0 (66.3)	2.8 (78.9)	0.3 (61.1)	0.2 (37.7)	2.0 (7.3)	2.5 (20.8)
**i.6**	42.3 (0.0)	6.0 (0.0)	8.2 (66.5)	2.1 (79.4)	0.4 (61.0)	0.2 (38.8)	2.2 (7.9)	2.7 (21.4)
**i.7**	39.8 (0.0)	7.8 (0.0)	7.1 (68.4)	2.2 (80.0)	0.6 (62.8)	0.5 (45.2)	3.8 (8.7)	3.7 (22.4)
**i.8**	39.0 (0.0)	8.2 (0.0)	6.7 (67.5)	1.8 (79.9)	0.6 (63.9)	0.4 (46.6)	4.2 (8.9)	3.9 (22.7)
**i.9**	36.6 (0.0)	13.7 (0.0)	6.3 (69.0)	2.1 (82.1)	1.2 (67.8)	0.9 (54.8)	8.7 (10.2)	7.8 (23.4)
**i.1** (n = 110)	31.2 (0.0)	6.6 (0.0)	3.1 (67.1)	0.3 (78.8)	0.8 (36.8)	0.4 (21.0)	3.0 (4.5)	3.8 (8.4)
**i.2**	34.2 (0.0)	8.4 (0.0)	4.5 (67.0)	1.0 (79.3)	1.1 (50.7)	0.8 (33.4)	4.6 (5.2)	5.5 (9.7)
**i.3**	35.0 (0.0)	6.3 (0.0)	4.4 (66.4)	0.8 (78.2)	0.5 (49.8)	0.4 (29.3)	2.9 (5.1)	3.7 (9.7)
**i.4**	36.5 (0.0)	4.8 (0.0)	4.3 (65.1)	1.0 (76.2)	0.7 (43.1)	0.3 (22.7)	2.3 (3.8)	3.0 (8.4)
**i.5**	38.3 (0.0)	4.6 (0.0)	5.8 (66.3)	1.0 (77.7)	0.5 (45.6)	0.3 (21.3)	2.0 (3.3)	2.6 (9.1)
**i.6**	36.7 (0.0)	4.1 (0.0)	4.3 (65.8)	0.6 (77.6)	0.5 (45.1)	0.3 (21.5)	1.5 (3.2)	2.2 (8.1)
**i.7**	34.5 (0.0)	6.3 (0.0)	4.6 (68.0)	0.7 (79.4)	1.0 (44.6)	0.5 (25.9)	3.0 (4.1)	3.9 (9.1)
**i.8**	33.4 (0.0)	6.4 (0.0)	3.6 (64.1)	0.5 (75.5)	0.7 (44.2)	0.5 (26.9)	3.0 (4.6)	3.9 (8.7)
**i.9**	31.3 (0.0)	9.9 (0.0)	3.4 (68.0)	0.7 (82.1)	1.3 (48.9)	1.1 (34.0)	5.9 (5.4)	6.8 (9.7)
**i.1** (n = 500)	15.0 (0.0)	2.1 (0.0)	0.7 (50.1)	0.0 (67.0)	0.5 (14.3)	0.2 (5.5)	0.7 (1.4)	1.2 (2.8)
**i.2**	17.6 (0.0)	2.2 (0.0)	1.3 (52.0)	0.2 (64.4)	0.7 (21.0)	0.3 (8.3)	0.9 (1.6)	1.5 (3.5)
**i.3**	19.5 (0.0)	2.3 (0.0)	1.2 (55.3)	0.2 (67.4)	0.5 (21.2)	0.3 (7.8)	0.8 (1.7)	1.2 (3.7)
**i.4**	22.5 (0.0)	1.8 (0.0)	1.8 (55.1)	0.1 (67.7)	0.4 (22.2)	0.2 (7.4)	0.8 (1.3)	1.3 (3.4)
**i.5**	25.0 (0.0)	1.7 (0.0)	2.2 (56.1)	0.3 (66.4)	0.4 (25.0)	0.2 (7.5)	0.5 (1.4)	1.2 (3.8)
**i.6**	22.8 (0.0)	1.8 (0.0)	1.8 (54.3)	0.1 (65.9)	0.3 (22.0)	0.2 (6.3)	0.5 (1.2)	1.1 (3.6)
**i.7**	18.4 (0.0)	2.0 (0.0)	1.5 (55.3)	0.1 (71.7)	0.7 (18.8)	0.3 (6.9)	0.9 (1.2)	1.5 (3.5)
**i.8**	18.3 (0.0)	2.1 (0.0)	1.0 (53.4)	0.0 (64.0)	0.4 (18.5)	0.2 (6.7)	0.7 (1.6)	1.5 (3.3)
**i.9**	13.4 (0.0)	3.0 (0.0)	0.8 (49.6)	0.1 (64.9)	0.6 (17.4)	0.4 (8.5)	1.2 (2.0)	1.5 (3.3)

Data represent percentage of wrong sign of the estimated regression coefficients $\left(\hat{\beta }\right)$ for n = 50, 110 and 500. The probability that a variable was not selected is given in parentheses. Ordinary least square estimation (OLS); Stepwise-backward variable selection with AIC (sAIC) and BIC (sBIC); Penalized estimation by using the Ridge method (Ridge), the least absolute shrinkage and selection operator (LASSO) and the elastic net L1-L2 (Enet 1), elastic net L2-L1 (Enet 2), elastic net L1+L2 (Enet 3).

### Scenario C

**Fig 6:**
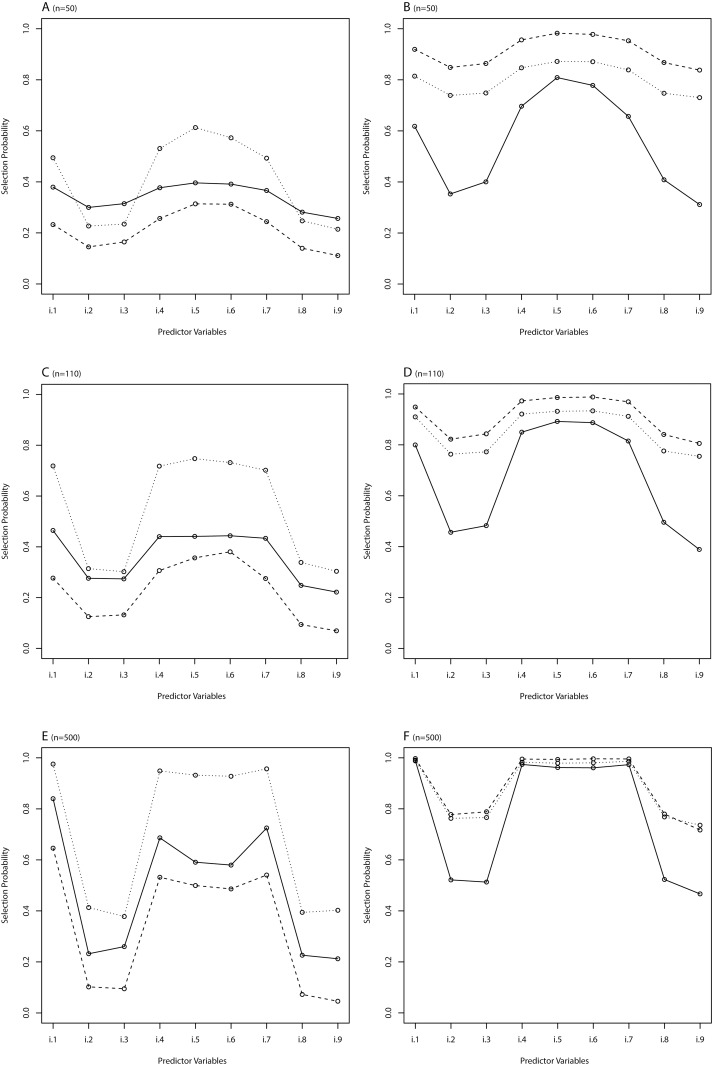
Scenario C: Selection probability for different variable selection methods in scenario C. n = 50 (A, B), n = 110 (C, D) and n = 500 (E, F). A, C, E: solid line for sAIC, dashed line for sBIC, dotted line for LASSO; B, D, F: solid line for Enet 1, dashed line for Enet 2, dotted line for Enet 3. Noise predictor variables are i.2, i.3, i.8, i.9.

This scenario was mainly focused to compare the performance of variable selection in the different methods with correlated noise variables (in contrast to scenario A, where the noise variable was uncorrelated to other exploratory variables). In general, LASSO type penalization (LASSO and Enet 1) showed an acceptable discrimination between parameters with true effect and noise variables as visualized in Fig 6. Particularly, type 1 error was improved by LASSO and Enet 1 (as compared to Enet 2 and Enet 3) in all sample size scenarios. As compared to sAIC and particularly sBIC, LASSO and Enet 1 showed higher type 1 error, but also tended to select variables with true effect more often. Concerning discrimination, LASSO type penalization showed improved results when the sample size was small (Fig 6A and 6B). However, it has to be mentioned that none of the strategies were able to derive suitable models in scenarios with small sample size and increasing correlation of independent variables. Thus LASSO was only superior over other less suitable approaches.

The model complexity (average number of included parameters in m = 5000 simulations) for Scenario B and C is given in Table 4: sBIC provided the sparsest models in both scenarios, followed by sAIC and LASSO (number of parameters in the model was twice as high as sBIC) and Enet 1. An optimal model in scenario C would contain the 5 variables with an effect or due to correlated variables even exclude some of these 5 variables. Of the model approaches with variable selection LASSO and Enet 1–3 produce rather too complex models, but on the other hand variables with a real effect are included more often. In sBIC with n = 50 and 110 on average 1.9–2.0 variables are included with seems too sparse. Of note, the average number of nuisance variables was approximately 32% for n = 50, however decreased with ascending sample size (approximately 20% for n = 110 and 10% for n = 500).

**Table 4:** Model complexity.

	OLS	sAIC	sBIC	Ridge	LASSO	Enet 1	Enet 2	Enet 3
**Scenario B**								
**n = 50**	9.0	3.0	1.8	9.0	3.3	4.9	8.2	7.0
**n = 110**	9.0	3.0	2.0	9.0	4.9	6.6	8.6	8.2
**n = 500**	9.0	4.2	3.0	9.0	7.2	8.4	8.9	8.7
**Scenario C**								
**n = 50**	9.0 (4.0)	3.1 (1.2)	1.9 (0.6)	9.0 (4.0)	3.6 (0.9)	5.0 (1.5)	8.2 (3.4)	7.2 (3.0)
**n = 110**	9.0 (4.0)	3.2 (1.0)	2.0 (0.4)	9.0 (4.0)	4.9 (1.3)	6.1 (1.8)	8.2 (3.3)	7.7 (3.1)
**n = 500**	9.0 (4.0)	4.4 (0.9)	3.0 (0.3)	9.0 (4.0)	6.3 (1.6)	6.9 (2.0)	8.0 (3.1)	8.0 (3.0)

Data represent the estimated (mean) number of included variables in m = 5000 simulations for n = 50, 110 and 500 with 9 and 5 meaningful variables and 0 and 4 nuisance variables for scenario B and C, respectively. For scenario C the average numbers of included nuisance variables are given in parentheses. Ordinary least square estimation (OLS); Stepwise-backward variable selection with AIC (sAIC) and BIC (sBIC); Penalized estimation by using the Ridge method (Ridge), the least absolute shrinkage and selection operator (LASSO) and the elastic net L1-L2 (Enet 1), elastic net L2-L1 (Enet 2), elastic net L1+L2 (Enet 3).

## Discussion

This report examines different model building strategies for predicting insulin sensitivity with nonorthogonal regressors. Particular focus was set to assess characteristics of penalized regression techniques, as compared to commonly used sequential variable selection to introduce these novel techniques in clinical investigations of diabetes with low dimensional data settings. Thereby, a possible question of research might be the creation of a surrogate index of insulin sensitivity. For this purpose, the results of our simulations (scenario A) showed that all investigated shrinkage strategies (i.e. Ridge, LASSO, as well as the elastic net regulations) moderately decreased the model prediction error of the final model as compared to the naïve OLS method or optimizing AIC or BIC with stepwise-backward selection. Recently, Ambler et al. [11] remarked, that penalized regression techniques offered improved prediction error and calibration as compared to standard methods in proportional hazard models with low events per variable. Consistently with our results, the improved effect of shrinkage procedures on the prediction error was mostly observed in the scenario with n = 50 observations and diminished with increasing sample size in our study, while the prediction error between different penalized estimation techniques was almost comparable in all scenarios. This observation is in accordance with Porzelius et al. [12], who found no large differences regarding the predictive performance between different shrinkage (and boosting) techniques in the setting of low dimensional survival studies.

However, further issues have to be considered in addition to prediction error in the model building process [19]. With an increasing number of covariates a prediction model gets unpractical for clinical or scientific use [25]. Therefore, model complexity is another major request for model building strategies. In our study Enet 2 (i.e. first optimizing *λ*_2_ followed by optimizing *λ*_1_) performed almost no variable selection mimicking pure Ridge type penalization, also reflected by the distribution of the shrinkage parameters. In contrast, LASSO and Enet 1 (i.e. first optimizing *λ*_1_ followed by optimizing *λ*_2_) performed much sparser and thus more parsimonious models particularly in scenarios with low (n = 50) and moderate (n = 110) sample size. Furthermore, amount of shrinkage (i.e. the size of the tuning parameters) strongly depends on the underlying amount of explained variation. As a consequence, type 1 error was considerably increased for LASSO type penalization in scenario A with n = 500, where explained variation was approximately 53%. In contrast type 1 error was much smaller in scenario C with an underlying explained variation of 31%. It was previously noticed, that the selective performance of LASSO is not invariant to the respective number of observations, as in case of high dimensional data scenarios the solution saturates after selecting a number of predictors comparable to the number of observations [9, 26, 27]. This limitation in addition to the observation that LASSO lacks to deal with grouped variables (i.e. tend to select one out of a group of highly correlated predictors and ignores others) motivated Zou and Hastie to introduce the Enet algorithm, which was supposed to deliver better results in these situations [9]. Hence, the capacity of Enet regulation to perform grouped selection might explain the behaviour of simultaneous *λ*_1_ and *λ*_2_ optimization (Enet 3) as it includes almost all of the highly correlated predictors in scenario A or moderately correlated predictor and noise variables in scenario C. Particularly, the higher type 1 error of these methods and a corresponding higher model complexity should be considered when Enet 2 and Enet 3 (or Ridge) are used for model building purposes. Therefore, LASSO type penalization outperforms Enet 2 or Enet 3 if the aim is to select some most relevant OGTT measurements. With regard to sequential strategies it has to be mentioned, that particularly sBIC performs sparser models as compared to sAIC or penalized techniques in almost all situations. The observed differences between sBIC and sAIC correspond to their different penalties: sBIC strongly depends on the number of observations in contrast to sAIC and therefore selects sparser models if ln(n) exceeds 2 (i.e. if n>7). Consequently, sBIC tends to select the correct model with infinite sample size and hence outperforms sAIC in such a situation (which tends to select too complex models), however with the limitation that sBIC chooses models which are too simple in scenarios with finite sample size [4]. This is also the explanation for its considerable lack of power when sample size is low.

Moreover, the accuracy of estimated regression coefficients was studied in scenario B. This is of particular importance in studies of carbohydrate metabolism for example if a researcher is aiming to evaluate the effect of repeated measurements during a metabolic stress test like the OGTT (actually only fasting and 120 min post load levels are interpreted in clinical routine and hence it might be of interest to evaluate the impact of other time points of glucose measurements within this examination). Although the investigated estimation techniques had to deal with a smaller number of covariables and collinearity was less severe as compared to scenario A we noticed a considerable advantage of shrinkage procedures as compared to OLS or sequential selection, regardless of the number of observations. Particularly, Ridge and Enet 2 estimated the variable effects with lowest bias. Despite a higher selection probability as compared to variable selection strategies (i.e. sAIC and sBIC with low sample size), parameter estimates with the wrong sign appeared considerably less often for LASSO type penalization (i.e. LASSO and Enet 1). This has to be considered as an important issue in modelling situations and in accordance with others we observed some major advantages for LASSO [28]: The analysis of our real data example (Fig 1) indicates that predictors of impaired insulin sensitivity (including OGTT derived measures) are usually positively correlated, resulting in regression coefficients with opposite signs if they are included into a multivariate model. This might cause major problems for interpreting variable specific effects, which could be avoided by variable selection and particularly by LASSO type penalisation if sample size is low. Moreover, some researchers have previously discussed a possible advantage of parameterwise shrinkage after backward elimination [29, 30]. Recently van Houwelingen and Sauerbrei [31] recommended this method, especially, when variable selection should be performed. We have not explicitly studied this two stage approach, which is quite different to the penalized least squares techniques (shrinkage during estimation) used in our study. However, the Enet 1 algorithm might be considered as a different two stage approach with LASSO type penalization for variable selection (first step) followed by Ridge regulation (second step), achieving the advantages of both strategies (variable selection, correct sign as well as low prediction error for parameter estimates). As sequential elimination might be inefficient in scenarios with few observations (n = 50) what was also indicated by the results of scenario C (where sAIC and sBIC showed inferior discrimination between noise variables and variables with true effect), we suggest that Enet 1 might provide improved results particularly if the sample size is low, predictors are correlated, and both–variable selection and accuracy of estimated coefficients are of interest. Thereby, the amount of selection of LASSO type penalization is closely related to the size of *λ*_1_. As the cross-validation process for optimizing (and defining) the tuning parameter obviously depends on the amount of the explained variation of the model this has additional impact on the selection behavior of LASSO and Enet 1.

Limitations of our study design have to be considered: In this study we aimed to introduce the use of variable shrinkage methods for clinical investigations in metabolic studies. Hence, the motivation of our simulation scenarios was rather to give some examples and our simulation scenarios are motivated (and restricted) by the key questions raised by our real data example due to correlated covariables. Moreover, it has to be mentioned, that there might be other methods, which might be used to deal with these problems like orthogonal transformation by (sparse) principal components analysis, which we have recently proposed for the analysis of OGTT data in another report [32].

We conclude, that there is not one “best” method, achieving superior performance over other strategies in every situation. There might be scenarios favoring different shrinkage or selection strategies depending on the correlation structure of predictors, the number of noise variables, the number of observations and particularly the question of research. With respect to the diabetes data example we draw the following conclusions: Prediction error is rather a consequence of sample size. However, as LASSO and Enet 1 derived sparser models as compared to Ridge, Enet 2 or Enet 3 and additionally resulted in a slightly improved prediction error as compared to sequential selection strategies, we recommend both methods for modelling insulin sensitivity in sparse sample size scenarios. Particularly smaller and hence simpler models might be more favourable for use in clinical setting. However, it has to be mentioned, that we found little to no improvement of penalized regression techniques over sequential OLS methods, when only prediction is the aim and the ratio between number of predictor variables and sample size of the study population is low. Furthermore, there is less advantage of the use of sequential selection methods in situations where the specific effect of correlated predictors is of interest and parsimonious but, however, meaningful models should be established. This is a common case in metabolic studies, where several correlated measurements over time are related to insulin sensitivity, whereby the most promising are candidates for use in clinical routine. The improvement in the accuracy of estimated effects as well as of their properties particularly for estimating the correct direction of parameters underlines the possible advantages of using penalized regression techniques for such tasks. However, the decision which procedure should be used depends on the specific context of a study and should involve clinical prior knowledge [28].
